# Multidisciplinary Management of Hepatocellular Carcinoma in Clinical Practice

**DOI:** 10.1155/2014/806391

**Published:** 2014-05-08

**Authors:** Gemma Bruera, Katia Cannita, Aldo Victor Giordano, Rosa Manetta, Roberto Vicentini, Sergio Carducci, Patrizia Saltarelli, Nerio Iapadre, Gino Coletti, Corrado Ficorella, Enrico Ricevuto

**Affiliations:** ^1^Medical Oncology, S. Salvatore Hospital, University of L'Aquila, 67100 L'Aquila, Italy; ^2^Department of Biotechnological and Applied Clinical Sciences, University of L'Aquila, 67100 L'Aquila, Italy; ^3^Radiology, S. Salvatore Hospital, 67100 L'Aquila, Italy; ^4^Hepatobiliar-Pancreatic Surgery, S. Salvatore Hospital, 67100 L'Aquila, Italy; ^5^Gastroenterology, S. Salvatore Hospital, 67100 L'Aquila, Italy; ^6^Infective Disease, S. Salvatore Hospital, 67100 L'Aquila, Italy; ^7^Pathology, S. Salvatore Hospital, 67100 L'Aquila, Italy

## Abstract

*Background*. Hepatocellular carcinoma (HCC) patients require different treatment strategies according to disease extension, liver function, and patient's fitness. We evaluated HCC multidisciplinary management in clinical practice. *Methods*. Consecutive patients were followed and treated with tailored medical, locoregional, and surgical treatments, according to disease stage and patient's fitness (age, Cumulative Illness Rating Scale (CIRS)). Activity, efficacy, and safety were evaluated. *Results*. Thirty-eight patients were evaluated: median age, 74; elderly 92%; CIRS secondary 28 (74%); Child-Pugh A 20 (53%), B 11 (29%); and Barcelona Clinic Liver Cancer (BCLC) 0 2 (5%), A 9 (24%), B 10 (26%), C 13 (34%), and D 4 (11%). Overall survival (OS) was 30 months. At 9 months median follow-up, among 25 unresectable HCC, OS was 10 months; BCLC B–D unfit for sorafenib showed OS 3 months. Ten patients (40%) received sorafenib: Child-Pugh A 5 (50%) and B 5 (50%) and disease control rate 89%, progression-free survival 7 months, and OS 9 months. G3-4 toxicities: anorexia, hypertransaminaemia, hyperbilirubinemia, and hypercreatininemia. Limiting toxicity syndromes were 40%, all multiple sites. *Conclusion*. HCC patients require multidisciplinary clinical management to properly select tailored treatments according to disease stage, fitness, and liver function. Patients suitable for sorafenib should be carefully selected, monitored for individual safety, and prevalently characterized by limiting toxicity syndromes multiple sites.

## 1. Introduction

Hepatocellular carcinoma (HCC) is a highly aggressive disease; only 10–20% of patients are candidates for curative surgery. In Western countries, the disease is diagnosed at early stages in 30–40% cases and is amenable to potentially curative treatments, such as surgical resection and liver transplantation and locoregional radiofrequency ablation [1]. Therapeutic options are stage dependent. Five-year survival up to 60–70% can be achieved in selected patients [1]. Reasons for tumor unresectability include coexisting advanced cirrhosis, large primary lesion, multifocal disease, invasion and thrombosis of major blood vessels, poor hepatic reserve, and extrahepatic metastases. Disease that is diagnosed at an advanced stage or progressing after locoregional therapy has a dismal prognosis, owing to the underlying liver disease and lack of effective treatment options. Approximately 80% have unresectable tumors, and the prognosis is very poor, with a median survival of only 4 months [2]. Treatment options for unresectable HCC may include locoregional [3–9] and systemic [10] therapy. Transarterial chemoembolization can increase survival in randomized studies, in a minority of patients. Thus, for the majority of HCC patients with unresectable tumors, best supportive care and systemic chemotherapy remain the main options for palliative treatment.

First line monochemotherapy, or more intensive regimens, reported overlapping activity and efficacy in phase III trials, ranging between objective response rate (ORR) 10–20.9% and overall survival (OS) 4–8.7 months [11, 12]. Conventional cytotoxic chemotherapy has not provided clinical benefit or prolonged survival for patients with advanced HCC [13]. Anthracyclines (i.e., doxorubicin) have been the most effective drugs, yielding up to 20% ORR, and 4 months median OS [11]. Cisplatin, interferon, doxorubicin, and fluorouracil (PIAF) used in combination showed promising activity in a phase II study. In a phase III randomized trial, median OS of the doxorubicin and PIAF arms was 6.8 months and 8.7 months (*P* = 0.83) and ORR 10.5% and 20.9%, respectively, not significantly different. Neutropenia, thrombocytopenia, and hypokalemia were significantly more common in patients treated with PIAF [12].

Cellular signalling mediated by the RAF-1 and vascular endothelial growth factor (VEGF) pathways has been implicated in the pathogenesis of HCC [14–17]. Sorafenib is a multikinase inhibitor that targets the RAF/MAP/ERK signalling pathway: it inhibits the serine-threonine kinases RAF-1 and B-RAF and the receptor tyrosine kinase activity of VEGF receptors (VEGFRs) 1, 2, and 3 and platelet-derived growth factor receptor *β* (PDGFR-*β*); it also targets KIT, FTL-3, and RET [18, 19]. In a mouse xenograft model of human HCC, sorafenib showed antiproliferative activity in liver-cancer cell lines, reduced tumor angiogenesis and tumor-cell signalling, and increased tumor-cell apoptosis [20].

Sorafenib was the first agent significantly increasing clinical outcome of advanced HCC [21–23]. A phase 2 study enrolling advanced HCC and Child-Pugh class A or B status indicated a median OS 9.2 months and a median time to progression 5.5 months [21]. Grade 3/4 drug-related toxicities included fatigue (9.5%), diarrhea (8.0%), and hand-foot skin reaction (5.1%). Two phase III trials reported progression-free survival (PFS) 2.8–5.5 months and OS 6.5–10.7 months [22, 23]. Advanced HCC patients, mostly Child-Pugh A, were randomly assigned to sorafenib or placebo. In the SHARP trial, >90% ECOG performance status 0-1 were enrolled and showed median OS significantly longer compared to placebo arm, 10.7 months versus 7.9 months (hazard ratio 0.69); the median time to symptomatic progression did not differ significantly (4.1 and 4.9 months, resp.);  median time to radiologic progression was significantly different (5.5 months and 2.8 months, resp.). ORR in the sorafenib group was 2%; disease control rate was significantly higher (43% versus 32%, resp.). In the Asia-Pacific phase III trial [23], patients were more likely to be younger, with HBV-related disease, symptomatic disease, and a higher number of tumor sites. Median OS and median time to progression were lower in both treatment and placebo groups (6.5 versus 4.2 months and 2.8 versus 1.4 months, resp.), even if significantly different (hazard ratio 0.68 and 0.57, resp.).

Clinical management of HCC faces with different options of treatment according to extension of disease, liver functional stage (Child-Pugh class), and patients' fitness (age, performance status (PS), and comorbidities). In clinical practice, a decision-making process including nutritional, functional, and comorbidity status is required to tailor first line medical treatment with sorafenib in the advanced stage. Elderly HCC patients are prevalent, and primary clinical challenge is proper selection of tailored treatments, according to prognostic factors, and by weighing expected safety and efficacy. Elderly status (age > 65 years), PS > 2, and/or comorbidities represent major features determining toxicities and limiting quality of life of treated patients, thus limiting indication of sorafenib.

Here we report an experience of multidisciplinary management and selection of tailored multimodality treatment options of consecutive HCC patients in clinical practice, according to defined clinical criteria.

## 2. Materials and Methods

### 2.1. Patient Eligibility

Consecutive HCC patients were evaluated by a multidisciplinary disease management team and treated in clinical practice with medical, locoregional, and/or surgical treatments, chosen among those in indication and approved for HCC treatment in different stages. Thus, it was not a clinical trial and any approval by ethics committee and institutional review board was not necessary, because patients were treated with conventional treatments without any additional medical intervention out of the best common clinical practice. Patients had radiological and/or histologically confirmed diagnosis of HCC, age ≥ 18 years. Patients were classified according to Cumulative Illness Rating Scale (CIRS) [24], Child-Pugh score, and Barcelona Clinic Liver Cancer (BCLC) stage. Treatment options were tailored according to age (< or ≥75 years) and patient's fitness (PS, CIRS). Patients with PS 3 were not treated with sorafenib. Criteria to define patients unfit for standard treatment strategies were uncontrolled severe diseases; cardiovascular disease (uncontrolled hypertension, uncontrolled arrhythmia, and ischemic cardiac diseases in the last year); thromboembolic disease, and coagulopathy, preexisting bleeding diatheses.  All patients provided written informed consent to the proposed treatment option.  All patients were registered in HCC registry of Regione Abruzzo, Italy (Hepaca registry), active from September 2010.

### 2.2. Methods

#### 2.2.1. Treatment Strategies

Medical treatment included sorafenib 400–800 mg/die orally administered, according to patient's fitness. Locoregional therapy was the conventional transarterial chemoembolization (cTACE), with administration of doxorubicin 30–50 mg.

#### 2.2.2. Study Design

Activity and efficacy were evaluated. Clinical evaluation of response was made by CT scan; RMN was added based on investigators' assessment. Patients were evaluated at baseline, and every two-three months by a multidisciplinary team, consisting of medical oncologist, radiologist, interventional radiologist, hepatobiliary-pancreatic surgeon, gastroenterologist, infectivologist, and pathologist, sharing and dynamically evaluating common multidisciplinary treatment strategies. Follow-up was scheduled every two-three months up to disease progression or death.

Toxicity was registered according to National Cancer Institute Common Toxicity Criteria (version 4.0). Limiting toxicity (LT) was defined as grade 3-4 nonhematologic toxicity, grade 4 hematologic toxicity, febrile neutropenia, or any toxicity determining >2 weeks delay of medical treatment. To discriminate individual safety, limiting toxicity syndromes (LTS), consisting of at least a LT associated or not to other limiting or G2 toxicities, were evaluated, as previously reported [25, 26]. LTS were classified into limiting toxicity syndromes single site (LTS-ss), characterized only by the LT, and limiting toxicity syndromes multiple sites (LTS-ms), characterized by ≥2 LTs or a LT associated to other, at least G2, nonlimiting toxicities.

Clinical criteria of activity and efficacy were ORR, PFS, and OS: ORR, evaluated according to RECIST criteria [27] and PFS and OS, evaluated using the Kaplan-Meier method [28]. PFS was defined as the length of time from the beginning of treatment and disease progression or death (resulting from any cause) or to the last contact and OS as the length of time between the beginning of treatment and death or to last contact. Log-rank test was used to compare OS [29].

## 3. Results

### 3.1. Patient Demographics

From September 2010 to October 2013, 38 new patients were evaluated by the HCC multidisciplinary team at S. Salvatore Hospital, L'Aquila, Italy. Clinical features of patients were (Table 1): male 79%; median age, 74 years; young-elderly 47% and old-elderly 45%; PS 0-1 81%. CIRS stage: primary 5%, intermediate 21%, and secondary 74% (28 patients). Underlying liver disease was hepatitis 24%, cirrhosis 63% (24 patients) and HCV-related 34% and alcoholic 39%. Complications of liver disease were thrombosis 18%, splenomegaly 37%, and varices 31.5%; altered baseline laboratory tests were thrombocytopenia 16% (6 patients), hypertransaminasemia 58%, cholestasis 63%, and hyperbilirubinemia 29%. Involved tumoral sites other than liver were observed in 37% (14 patients): metastatic sites were lung 8%, lymph nodes 29%, bone 8%, ascites 24%, and pleural effusion 5%. Liver nodules were single 24% (9 patients) and multiple 76% (29 patients). Diagnosis of HCC was clinical in 24 patients (63%) and histopathological in 14 (37%). Previous therapies were surgery 9 patients (24%) and TACE 10 (26%). At the time of the multidisciplinary team evaluation: alphafetoprotein negative 17 (48%), positive 3 (8%), ≥200 ng/mL 10 (26%), unknown 8 (21%); Child-Pugh score A 20 (53%), B 11 (29%), and C 3 (8%); BCLC stage 0 2 (5%),  A 9 (24%),  B 10 (26%),  C 13 (34%),  D 4 (11%). Treatment choices were: follow-up in 11 patients (29%),  surgery 1 (2%),  biopsy 3 (8%),  TACE 5 (13%),  sorefenib 12 (32%), and best supportive care 6 (16%).

Twenty-five HCC patients (66%) showed unresectable HCC or advanced/metastatic HCC: 10 (40%) were treated with sorafenib; 15 (60%) were unfit for treatment with sorafenib due to age and/or CIRS and/or performance status 3,  liver functional status, and altered laboratory tests.

### 3.2. Demographics of Patients Treated with Sorafenib

Clinical features of the 10 patients treated with sorafenib were (Table 1): median age, 73 years; young- and old-elderly, 4 (40%) and 5 (50%), respectively; PS 0, 4 (40%), 1, 6 (60%); and  CIRS stage intermediate 2 (20%),  secondary 8 (80%). Baseline altered laboratory tests were thrombocytopenia 3 (30%), transaminase elevation 7 (70%), cholestasis 10 (100%), and hyperbilirubinemia 5 (50%). Involved tumoral sites were 1 in 4 patients (40%) and ≥2 in 6 (60%); metastatic sites were lung 1 (10%), lymph nodes 5 (50%), bone 2 (20%), and ascites 3 (30%); liver nodules were multiple in all patients. Diagnosis of HCC was clinical in 6 patients (60%) and histopathological in 4 (40%). Previous locoregional treatments (TACE) were performed in 3 patients (30%). At the time of the multidisciplinary team evaluation alphafetoprotein was negative 2 (20%), positive 2 (20%), and ≥200 ng/mL 6 (60%); Child-Pugh score was A 5 (50%)  and B 5 (50%);  BCLC stage was B 3 (30%), C 6 (60%), and D 1 (10%). Sorafenib was administered in selected patients (Child-Pugh A/B and BCLC stage B–D) at different doses, according to age and CIRS stage: 800 mg/die in 3 patients (30%),  600 mg/die 3 (30%),  and 400 mg/die 4 (40%).

### 3.3. Overall Activity and Efficacy

**Figure 1:**
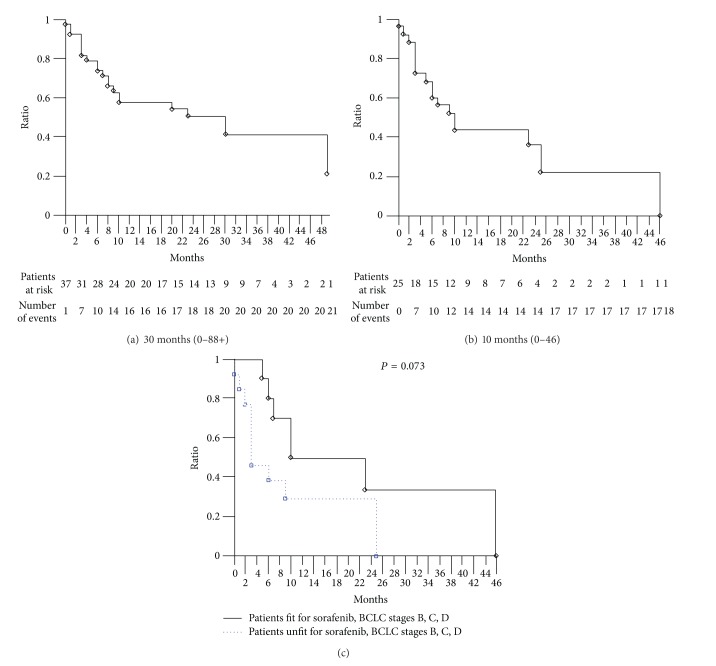
Kaplan-Meier survival estimate. (a) Overall hepatocellular carcinoma patients, overall survival. (b) Unresectable hepatocellular carcinoma patients,  overall survival. (c) Unresectable hepatocellular carcinoma patients, BCLC stages B, C,  and D,  fit versus unfit for sorafenib.

Among the overall 38 patients, at a median follow-up of 15.5 months, median OS was 30 months (0–88+): 21 events occurred (Figure 1(a)). Among 25 unresectable HCC, at a median follow-up of 9 months, median OS was 10 months (0–46): 18 events occurred (72%) (Figure 1(b)). Among 15 patients with unresectable HCC or with advanced/metastatic HCC unfit for treatment with sorafenib, median OS was 6 months (0–40+): 11 events occurred (73%). In this subgroup, BCLC B, C, and D HCC patients showed median OS 3 months (0–25), trendily worse compared with BCLC B,  C,  and D HCC patients treated with sorafenib (*P* = 0.073) (Figure 1(c)).

### 3.4. Sorafenib Activity and Efficacy

**Figure 2:**
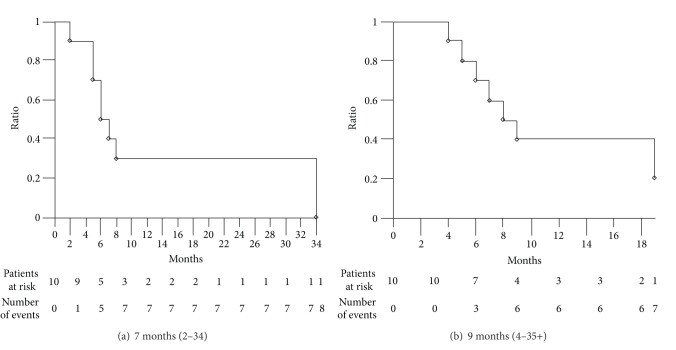
Kaplan-Meier survival estimate. Patients treated with sorafenib. (a) Progression-free survival. (b) Overall survival.

Among the 10 patients who underwent medical treatment with sorafenib, 9 were evaluable for activity (Table 2). The intent-to-treat analysis showed 2 partial responses (22%), 6 stable diseases (67%), and 1 progressive disease (11%). Disease control rate was 89% (*α* 0.05, CI ± 22). Median PFS was 7 months (2–34): 8 events occurred (Figure 2(a)). Median OS was 9 months (4–35+): 7 events occurred (Figure 2(b)).

### 3.5. Dose-Intensity and Toxicity

Median number of cycles per patient was 4 (range 1–9). Median received dose intensity (rDI) per cycle was 315 (124–800) mg/die, 39% of the recommended dose.

G3-4 toxicities, by patients, in 45 cycles, were (Table 3) anorexia, 1 (10%); hypertransaminaemia, 4 (40%), hyperbilirubinemia,  1 (10%); and  hypercreatininemia, 2 (20%). G2 toxicities were anorexia 3 (30%), diarrhea 4 (40%), constipation 2 (20%), asthenia 5 (50%), epistaxis 2 (20%), hand-foot syndrome 1 (10%), hypertransaminaemia, 1 (10%), hyperbilirubinemia, 3 (30%), cholestasis 1 (10%), hypothyroidism 2 (20%), and thrombocytopenia 2 (20%). No case of thrombosis, hemorrhage/bleeding, cardiac or cerebrovascular ischemia, G4 neutropenia, febrile neutropenia, severe thrombocytopenia, or toxic deaths were observed. LTS were observed in 4 out of 10 patients (40%), all LTS-ms characterized by LT associated to other, at least G2, nonlimiting toxicities (Table 4). LTS were (Table 5) G2 thrombocytopenia for more than 2 weeks associated to G2 neutropenia; G2 hand-foot syndrome associated to G2 anorexia, G2 asthenia, and G2 hypothyroidism; G2 hyperbilirubinemia associated to G2 hypertransaminasemia; and G2 hyperbilirubinemia associated to G3 hypertransaminasemia, G4 cholestasis, G2 hypothyroidism, and G2 diarrhea.

## 4. Discussion

The present experience of clinical management of consecutive HCC patients by our multidisciplinary team in clinical practice showed that patients were mostly elderly (92%), equivalently young- and old-elderly, and prevalently with CIRS stage secondary (74%), PS 0-1 (81%), an underlying cirrhosis (63%), Child-Pugh A/B 82%, BCLC B-D 71%. Patients with unresectable or advanced/metastatic HCC were 66% mostly (60%) unfit for sorafenib, due to elderly and/or CIRS and/or performance status 3, altered liver functional status, and 40% fit for sorafenib. Median OS of unresectable or advanced/metastatic HCC was 10 months, and BCLC B–D patients unfit for sorafenib showed trendily worse prognosis (3 months).

HCC patients treated with sorafenib had PS 0-1, Child-Pugh class A and B, and received a median dose intensity 315 mg/die (39%). The present tailored approach, based on evaluation of elderly status and/or CIRS and functional liver status, as prevalently addressing selection of suitable patients and different doses of sorafenib, was associated with a disease control rate 89%, PFS 7 months and OS 9 months that requires further prospective validation. A complex decision-making process discriminating patients' fitness and tailoring medical treatment is challenging in this disease, mostly affecting elderly patients with comorbidities. Our experience of multidisciplinary and tailored clinical management is in agreement with prospective phase II/III trials evaluating sorafenib in different populations, mostly characterized by Child-Pugh class A, that reported significantly increased PFS 2.8–5.5 months and OS 6.5–10.7 months [21–23]. Subgroup analyses suggested that sorafenib is effective irrespective of the baseline ECOG PS (0–2), tumor burden (presence or absence of macroscopic vascular invasion and/or extrahepatic spread), presence or absence of either lung or lymph nodes metastasis, tumor stage, prior therapy, and disease etiology (alcohol-related or HCV-related HCC) [30, 31]. Child-Pugh class B benefited to the same extent as class A patients in terms of activity and PFS, but with lower OS [32] (3.2 versus 9.5 months) [33–39]. In a large retrospective study, median OS was 5.5 months compared to 11.3 months, respectively [35]. The prospective GIDEON trial confirmed that safety profile and time to progression of sorafenib was generally similar, and median OS was shorter in Child-Pugh class B patients [38, 40], 5.2 months compared to 13.6 months, respectively [40]. In Asian patients with advanced HBV-related HCC, there were no significant differences in clinical benefit, OS (5.5 versus 5 months), grade 3/4 hematologic toxicities.

Randomized studies showed that significantly prevalent grade 3/4 toxicities [22, 23] were weight loss, hand-foot skin reaction (5–11%), diarrhoea (6–8%) [22], hypophosphatemia (11%), thrombocytopenia (4%), and fatigue (3.4%). Hand-foot skin reaction and diarrhoea were the most common adverse events resulting in dose reductions. Sorafenib-associated adverse events led to dose reductions and interruptions in 26% and 44% of patients, respectively. Grade 3/4 adverse events in the Child-Pugh A and B groups, respectively, included hyperbilirubinemia (14% and 53%), ascites (3% and 5%), and encephalopathy (3% and 13%), and were more frequent in Child-Pugh class B subgroup [34]. In the GIDEON trial, the incidence of adverse events was similar across subgroups, although serious adverse events were more common in Child-Pugh class B patients [40]. In advanced HBV-related HCC, there were no significant differences in grade 3/4 hematologic toxicities and nonhematologic toxicities [41]. In our experience of tailored treatment according to patient's age and CIRS status and careful safety monitoring, cumulative G3-4 toxicities were anorexia (10%), hypertransaminasemia (40%), hyperbilirubinemia (10%), hypercreatininemia (20%). More, G2 diarrhea was observed in 40%. Elderly HCC patients treated with sorafenib showed 40% individual LTS, previously reported by our group as significantly more frequent in elderly metastatic colorectal cancer patients [25, 26]. LTS were almost exclusively characterized by LTS-ms, mainly including thrombocytopenia, hand-foot syndrome, liver dysfunction, anorexia, asthenia, hypothyroidism, and diarrhea.

cTACE can increase survival in selected patients with inoperable intermediate HCC (BCLC stage B) [1, 3], with 1-,  2-, and 3-year survival of 75%, 47%, and 26%, respectively [1]. After TACE, tumor microenvironment becomes deranged with increased hypoxia, leading to upregulation of hypoxia inducible factor-1*α*, which in turn upregulates VEGF and PDGFR and increases tumor angiogenesis [42, 43], that may have adverse protumor consequences [44, 45]. There has been interest in combining antiangiogenic targeted agents with TACE to decrease post-TACE angiogenesis, to improve the efficacy of locoregional therapy, and to decrease the incidence of systemic disease. Preclinical models combining transarterial embolization with antiangiogenic agents reported a reduction in tumor volume and vessel density, as well as a prolongation in survival compared with transarterial embolization alone [46]. In nonrandomized phase II studies, sorafenib concomitant with TACE or doxorubicin-eluting beads (DEB) TACE was well tolerated and effective in unresectable HCC [47–51]. In a phase III randomized trial, sorafenib when given after TACE did not significantly increase time to progression or OS in patients who responded to TACE, potentially due to delays in starting sorafenib after TACE (median 9 weeks) and/or low daily sorafenib doses [52].

The present experience based on multidisciplinary management in clinical practice of a small cohort of consecutive HCC patients in a single institution to select proper tailored treatment options, based on comorbidity and functional liver status, requires further validation in a wide population. Careful selection of HCC patients suitable for further development of therapeutic strategies in HCC, integrating sorafenib with TACE, and including innovative targeted agents, according to prognostic and predictive biomarkers, is mandatory.

## 5. Conclusion

In clinical practice, HCC patients require multidisciplinary clinical management and selection of tailored locoregional and medical treatments, according to disease stage and patient's age and comorbidities. HCC patients suitable for sorafenib treatment should be carefully selected and monitored for individual safety.

## Figures and Tables

**Table 1:** Patients' features.

	Overall patients	Unresectable HCC	Patients treated with sorafenib	Patients untreated with sorafenib
	Total number (%)	Total number (%)	Total number (%)	Total number (%)
Number of patients	38	25 (66)	10 (40)	15 (60)
Sex				
Male/female	30/8	19/6	8/2	11/4
Age, years				
Median	74	74	73	74
Range	58–86	63–85	63–80	65–85
Elderly				
≥65 <75 years	18 (47)	12 (48)	4 (40)	8 (53)
≥75 years	17 (45)	11 (44)	5 (50)	6 (40)
WHO performance status				
0	10 (26)	6 (24)	4 (40)	2 (13)
1	21 (55)	13 (52)	6 (60)	7 (47)
2	6 (16)	5 (20)	—	5 (33)
3	1 (3)	1 (4)	—	1 (7)
CIRS stage				
Primary	2 (5)	2 (8)	—	2 (13)
Intermediate	8 (21)	4 (16)	2 (20)	2 (13)
Secondary	28 (74)	19 (76)	8 (80)	11 (73)
Liver disease				
Hepatitis	9 (24)	5 (20)	2 (20)	3 (20)
Cirrhosis	24 (63)	18 (80)	7 (70)	11 (73)
Etiology				
HBV	1 (3)	1 (4)	1 (10)	—
HCV	13 (34)	9 (36)	2 (20)	7 (47)
HBV + HCV	1 (3)	—	—	—
Alcoholic	15 (39)	10 (40)	3 (30)	7 (47)
Idiopathic	8 (21)	3 (12)	2 (20)	1 (7)
Complications				
Varices	12 (31.5)	8 (32)	4 (40)	4 (27)
Thrombosis	7 (18)	5 (20)	1 (10)	4 (27)
Splenomegaly	14 (37)	12 (48)	6 (60)	6 (40)
Laboratory tests				
Thrombocytopenia	6 (16)	5 (20)	3 (30)	2 (13)
Hypertransaminasemia	22 (58)	19 (76)	7 (70)	12 (80)
Cholestasis	24 (63)	21 (84)	10 (10)	11 (73)
Hyperbilirubinemia	11 (29)	11 (44)	5 (50)	6 (40)
Number of involved sites				
1	24 (63)	11 (44)	4 (40)	7 (47)
≥2	14 (37)	14 (56)	6 (60)	8 (53)
Sites of metastases				
Lung	3 (8)	3 (12)	1 (10)	2 (13)
Lymph nodes	11 (29)	11 (44)	5 (50)	6 (40)
Bone	3 (8)	3 (12)	2 (20)	1 (7)
Ascites	9 (24)	8 (32)	3 (30)	5 (33)
Pleural effusion	2 (5)	2 (8)	—	2 (13)
Liver nodules				
Single	9 (24)	2 (8)	—	2 (13)
Multiple	29 (76)	23 (92)	10 (100)	13 (87)
Diagnosis				
Clinical	24 (63)	19 (76)	6 (60)	13 (87)
Histophatological	14 (37)	6 (24)	4 (40)	2 (13)
*α*fetoprotein at DMT evaluation				
Negative	17 (48)	6 (24)	2 (20)	4 (27)
Positive	3 (8)	4 (16)	2 (20)	2 (13)
≥200 ng/mL	10 (26)	13 (52)	6 (60)	7 (47)
Unknown	8 (21)	2 (8)	—	2 (13)
Child-Pugh score at DMT evaluation				
A	20 (53)	11 (44)	5 (50)	6 (40)
B	11 (29)	12 (48)	5 (50)	7 (47)
C	3 (8)	2 (8)	—	2 (13)
Unknown	4 (10)	—	—	—
BCLC stage at DMT evaluation				
0	2 (5)	—	—	—
A	9 (24)	2 (8)	—	2 (13)
B	10 (26)	7 (28)	3 (30)	4 (27)
C	13 (34)	13 (52)	6 (60)	7 (47)
D	4 (11)	3 (12)	1 (10)	2 (13)
DMT treatment choice				
Follow-up	11 (29)	2 (8)	—	2 (13)
Surgery	1 (2)	—	—	—
Biopsy	3 (8)	3 (12)	—	3 (20)
TACE	5 (13)	3 (12)	—	3 (20)
Sorafenib	12 (32)	12 (48)	10 (100)	2 (13)
Best supportive care	6 (16)	5 (20)	—	5 (33)
Sorafenib treatment				
800 mg/die	3 (8)	3 (30)	3 (30)	—
600 mg/die	3 (8)	3 (30)	3 (30)	—
400 mg/die	4 (10.5)	4 (40)	4 (40)	—

WHO: World Health Organization; CIRS: Cumulative Illness Rating Scale; TACE: transarterial chemoembolization; DMT: disease management team.

**Table 2:** Activity and efficacy of sorafenib.

	Intent-to-treat Analysis	
	Number	%
Enrolled patients	10	100
Evaluable patients	9	90
Objective response	2	22 (CI ± 29)
Partial response	2	22
Complete response	—	—
Stable disease	6	67
Progressive disease	1	11
Median progression-free survival, months	7	
Range	2–34	
Progression events	8	80
Median overall survival, months	9	
Range	4–35+	
Deaths	7	70

**Table 3:** Cumulative toxicity.

	Patients				Cycles			
Number	**10**				**45**			
NCI-CTC Grade	1	2	3	4	1	2	3	4
Nausea (%)	1 (10)	—	—	—	1 (2)	—	—	—
Vomiting (%)	1 (10)	—	—	—	1 (2)	—	—	—
Anorexia (%)	4 (40)	3 (30)	1 (10)	—	14 (31)	4 (9)	1 (2)	—
Diarrhea (%)	1 (10)	4 (40)	—	—	6 (13)	7 (16)	—	—
Hypoalbuminemia (%)	—	—	—	—	—	—	—	—
Constipation (%)	—	2 (20)	—	—	1 (2)	2 (4)	—	—
Stomatitis/mucositis (%)	2 (20)	—	—	—	12 (27)	—	—	—
Asthenia (%)	4 (40)	5 (50)	—	—	30 (67)	5 (11)	—	—
Hypertension (%)	1 (10)	—	—	—	1 (2)	—	—	—
Hypotension (%)	—	—	—	—	—	—	—	—
Gengival recession/gengivitis (%)	—	—	—	—	—	—	—	—
Rhinitis (%)	1 (10)	—	—	—	1 (2)	—	—	—
Epistaxis (%)	—	2 (20)	—	—	2 (4)	2 (4)	—	—
HFS (%)	—	1 (10)	—	—	3 (7)	1 (2)	—	—
Hyponatriemia (%)	2 (20)	—	—	—	2 (4)	—	—	—
Hypertransaminasemy (%)	3 (30)	1 (10)	2 (20)	2 (20)	14 (31)	13 (29)	2 (4)	1 (4)
Hyperbilirubinemia (%)	4 (40)	3 (30)	1 (10)	—	17 (38)	5 (11)	4 (9)	—
Cholestasis (%)	2 (20)	1 (10)	1 (10)	—	12 (27)	11 (24)	14 (31)	1 (2)
Hyperammoniemia (%)	3 (30)	—	—	—	5 (11)	—	2 (4)	—
Hypercreatininemia (%)	2 (20)	—	2 (20)	—	—	—	—	—
Hypothyroidism (%)	—	2 (20)	—	—	—	2 (4)	—	—
Anemia (%)	—	—	—	—	—	—	—	—
Leucopenia (%)	2 (20)	1 (10)	—	—	5 (11)	1 (2)	—	—
Neutropenia (%)	—	1 (10)	—	—	2 (4)	1 (2)	—	—
Thrombocytopeny (%)	4 (40)	2 (20)	—	—	8 (18)	6 (13)	—	—

**Table 4:** Distribution of limiting toxicity syndromes.

	Overall	
	No.	%
Patients	10	100
Limiting toxicity syndromes (LTS)	4	40
LTS single-site (LTS-ss)	—	—
LTS multiple-sites (LTS-ms)	4	40
Single LT plus G2-3	4	40
Double LTs	—	—

LT: limiting toxicity; G: grade.

**Table 5:** Limiting toxicity syndromes.

Patients #	Age (years)	LT	Associated toxicity	
			LT	G2-G3
1	70	Thrombocytopenia G2 > 2 weeks	—	Neutropenia G2

2	75	Hand-foot syndrome G2	—	Anorexia G2 Astenia G2 Hypothyroidism G2

3	71	Hyperbilirubinemia G2	—	Hypertransaminasemy G2

4	65	Hyperbilirubinemia G2	—	Hypertransaminasemy G3 Cholestasis G4 Hypothyroidism G2 Diarrea G2

LT: limiting toxicity; G: grade.
